# The clinical outcomes of cementless unicompartmental knee replacement in patients with reduced bone mineral density

**DOI:** 10.1186/s13018-020-1566-2

**Published:** 2020-01-31

**Authors:** Hasan R. Mohammad, James A. Kennedy, Stephen J. Mellon, Andrew Judge, Christopher A. Dodd, David W. Murray

**Affiliations:** 1 Nuffield Department of Orthopaedics, Rheumatology and Musculoskeletal Sciences University of Oxford, Nuffield Orthopaedic Centre, Oxford, OX3 7LD, UK; 2 Musculoskeletal Research Unit, Level 1 Learning and Research Building, University of Bristol, Southmead Hospital, Bristol, BS10 5NB, UK; 3 Nuffield Orthopaedic Centre, Oxford University Hospitals NHS Foundation Trust, Windmill Rd, Oxford, OX3 7LD UK

**Keywords:** Bone mineral density, Cementless, Oxford UKR

## Abstract

**Background:**

Osteoporosis and osteopenia are conditions characterised by reduced bone mineral density (BMD). There is concern that bone with reduced BMD may not provide sufficient fixation for cementless components which primarily rely on the quality of surrounding bone. The aim of our study was to report the midterm clinical outcomes of patients with reduced BMD undergoing cementless unicompartmental knee replacements (UKR). Our hypothesis was that there would be no difference in outcome between patients with normal bone and those with reduced BMD.

**Methods:**

From a prospective cohort of 70 patients undergoing cementless UKR surgery, patients were categorised into normal (*n* = 20), osteopenic (*n* = 38) and osteoporotic groups (*n* = 12) based on their central dual-energy X-ray absorptiometry (DEXA) scans according to the World Health Organization criteria. Patients were followed up by independent research physiotherapists and outcome scores; Oxford Knee Score (OKS), Tegner score, American Knee Society Score Functional (AKSS-F) and Objective (AKSS-O) were recorded preoperatively and at a mean of 4 years postoperatively. The prevalence of reoperations, revisions and mortality was also recorded at a mean of 5 years postoperatively.

**Results:**

There were no significant differences in the midterm postoperative OKS (*P* = 0.83), Tegner score (*P* = 0.17) and AKSS-O (*P* = 0.67). However, the AKSS-F was significantly higher (*P* = 0.04) in normal (90, IQR 37.5) compared to osteoporotic (65, IQR 35) groups. There were no significant differences (*P* = 0.82) between normal and osteopenic bone (80, IQR 35). The revision prevalence was 5%, 2.6% and 0% in the normal, osteopenic and osteoporotic groups respectively. The reoperation prevalence was 5%, 7.9% and 0% respectively. There were no deaths in any group related to the implant.

**Conclusions:**

We found that patients with reduced BMD could safely undergo cementless UKR surgery and have similar clinical outcomes to those with normal BMD. However, larger studies with longer follow-up are needed to confirm our findings and ensure that cementless fixation is safe in patients with reduced BMD.

## Background

Over 100,000 primary knee replacements are conducted annually in the UK for end-stage osteoarthritis [1]. These include total knee replacement (TKR) and unicompartmental knee replacement (UKR), where one compartment is replaced. Both TKR and UKR can be implanted with or without bone cement. Most knee replacement research has focused on implant design rather than the quality of bone that the implants are inserted into.

Osteoporosis and its milder form, osteopenia, are conditions characterised by reduced bone mineral density (BMD) in otherwise normal bones, rendering them more fragile and fracture prone [2]. They are estimated to affect over 200 million people worldwide costing 37 billion euros annually in Europe alone [3, 4]. Their prevalence is increasing as the population is ageing. Although osteoarthritis and reduced BMD have been considered to be mutually exclusive, recently, it has been shown that both conditions can coexist [5–7]. Current estimates are that approximately 10% of patients undergoing hip or knee replacement surgery have a diagnosis of osteoporosis and up to approximately 30% have evidence of osteopenia [5].

There are concerns that patients with reduced BMDs will suffer worse outcomes after joint replacement surgery [8, 9]. Surgeons are particularly concerned regarding cementless implants, given they rely on press fit fixation in the surrounding bone for primary stability [10]. Biomechanical studies using polyurethane bone analogues suggest that implant stability is directly related to BMD for cementless implants and where BMD is reduced, fixation is greatly enhanced by the addition of cement [11]. As a result, cementless implants are generally reserved for younger patients with higher BMDs [12]. Despite these concerns, there have been no studies investigating the clinical outcomes of patients with reduced BMDs.

Our study aim was to determine whether patients with reduced BMD have worse clinical outcomes following uncemented UKR than those with normal BMD. Our null hypothesis was that there would be no difference in outcome between patients with normal and low BMD. We report the midterm outcomes of a prospective cohort of normal, osteopenic and osteoporotic patients undergoing cementless UKR surgery.

## Methods

Between August 2006 and February 2017, a consecutive series of 70 patients with central dual-energy X-ray absorptiometry (DEXA lumbar spine, hip and femoral neck) as part of their routine clinical care underwent medial cementless Oxford Phase 3 UKR (Zimmer Biomet, Swindon, UK). The patients had DEXA scans because they were considered at risk of fragility fracture according to guidelines published by the National Institute of Clinical Excellence (NICE) and were not related to UKR surgery [13].

Surgery was performed through a minimally invasive approach by two surgeons involved in the design of the implant (DWM, CAD). The recommended indications and surgical technique were used [14, 15]. The indications for surgery were anteromedial osteoarthritis or avascular necrosis with functionally intact ligaments. Osteoporosis and osteopenia were not considered to be contraindications. The only exclusion criteria were patients without a DEXA scan.

Patients were categorised into three groups based on their *T* scores from their DEXA scans. From the three regions (lumbar spine, hip, femoral neck), the lowest mean *T* scores were used to diagnose patients as per WHO recommendations [16] into (1) normal bone, (2) osteopenic bone or (3) osteoporotic bone (Table 1).

**Table 1:** WHO definitions of normal bone, osteopenia and osteoporosis used to interpret central dual-energy X-ray absorptiometry scans

Bone status	*T* score
Normal	*T* ≥ − 1.0
Osteopenia	− 2.5 < *T* < − 1.0
Osteoporosis	*T* ≤ − 2.5

Baseline characteristics including age, body mass index (BMI), gender and American Society of Anaesthiologists (ASA) grade were recorded. All patients were followed up by research physiotherapists independent to the surgical and clinical teams caring for the patients by clinic appointments, postal questionnaires or telephone calls. Outcome scores including the Oxford Knee Score (OKS) [17], American Knee Society Objective Score (AKSS-O) [18], American Knee Society Functional Score (AKSS-F) [18] and the Tegner Activity Score [19] were recorded preoperatively and regularly postoperatively at a mean of 4 years. The AKSS-O was calculated, as previously described [20], without deductions if the alignment was not neutral, as the Oxford UKR does not aim to achieve neutral alignment like TKR, but aims to restore pre-disease alignment, which is commonly varus. The status of the replacements was assessed by a full-time research physiotherapist and data manager, by contacting patients directly and screening hospital and other records. The prevalence of revision, reoperation and patient mortality were recorded as they occurred. Revision was defined as the removal or addition of any implant component. Reoperation was defined any surgical intervention whatsoever to the knee and included all revisions.

All study data is recorded in an electronic database and updated in real time by a full time data manager and was extracted on 14^th^ August 2018.

### Statistical analysis

Non-parametric statistics were used to compare patient-reported outcome measures as these were not normally distributed.

To analyse differences in functional scores pre- and post-operatively, the Wilcoxon matched signed-rank test was utilised. To compare the postoperative scores between groups, the Kruskal-Wallis test was utilised. The post hoc Dunn test was utilised if the Kruskal-Wallis test approached statistical significance. The OKS functional and pain subscales were calculated according to the published recommendations [21]. The Kruskal-Wallis test was used to compare for baseline differences between groups (Table 2). The prevalence of reoperations, revisions and deaths are reported as a percentage of the total number of patients in that group.

**Table 2:** Comparisons of baseline characteristics between groups. The Kruskal-Wallis test was used to compare all variables except gender which was compared with the Fisher’s exact test. Mean (SD) and Median (IQR) values for patient reported outcome measures are shown

Parameter	Normal bone	Osteopenic bone	Osteoporotic bone	Differences between groups for parameter
Mean follow-up (years)	4.7 (SD 2.4)	4.7 (SD 2.5)	6.6 (SD 3)	*P* = 0.16
Age	62.9 (SD 8.1)	70.4 (SD 9.7)	68 (SD 8.3)	*P* = 0.01
BMI	30.9 (SD 6.3)	27 (SD 4.5)	27.1 (SD 5.7)	*P* = 0.06
Gender	15 females, 5 males	34 females, 4 males	11 females, 1 male	*P* = 0.34
ASA grade	2.25 (SD 0.6)	2.16 (SD 0.5)	2.17 (SD 0.4)	*P* = 0.76
Preop mean and median OKS	19.3 (SD 7.8), 17 (IQR 11)	23.1 (SD 8.8), 23.5 (IQR 15)	20.4 (SD 7.5), 20.5 (IQR 9)	*P* = 0.37
Preop mean and median OKS functional subscale score	10 (SD 3.8), 10 (IQR 6)	11 (SD 3.9), 11 (IQR 6)	11.3 (SD 3.7), 12 (IQR 6)	*P* = 0.58
Preop mean and median OKS pain subscale score	10.5 (SD 5), 10 (IQR 9)	11.1 (SD 5.1), 10 (IQR 7)	11.7 (SD 5.5), 13 (IQR 7)	*P* = 0.80
Preop mean and median Tegner	1.7 (SD 1.4), 2 (IQR 3)	1.9 (SD 0.9), 2 (IQR 2)	1.5 (SD 1), 1.5 (IQR 1)	*P* = 0.70
Preop mean and median AKSS-O	48.7 (SD 14.2), 50 (IQR 14)	58 (SD 12.8), 59.5 (IQR 16.5)	50 (SD 14.5), 48 (IQR 11)	*P* = 0.18
Preop mean and median AKSS-F	62.1 (SD 16.8), 62.5 (IQR 12.5)	65.1 (SD 14.3), 70 (IQR 15)	60 (SD 12.6), 60 (IQR 15)	*P* = 0.74

Statistical analyses were all performed in Stata version 14 (STATA Corp, TX). Statistical significance was set at *P* ≤ 0.05.

## Results

### Overall results from cohort

Seventy medial Oxford UKRs were implanted in 70 patients (60 females and 10 males). The indications for surgery were anteromedial osteoarthritis in 65 patients and spontaneous osteonecrosis of the knee in 5 patients. Thirty-six UKRs were right sided and 34 were left sided. The mean age at surgery was 68.0 years (SD 9.5) and mean BMI was 28.1 (SD 5.5).

The mean follow-up of patients for information about revision and reoperation 5 years (SD 2.6) and for post-operative scores was 4 years (SD 2.5). No patient was lost to follow-up, but one patient withdrew from regular follow-up due to poor general health after 7 years follow-up. From the 70 patients, 20 met the definition for normal bone, 38 had osteopenic bone and 12 had osteoporotic bone as per the WHO criteria [16]. There were no significant differences in the baseline demographics of the three groups except age (Table 2).

The mean and median preoperative OKS was 21.7 (SD 8.3) and 22 (IQR 13) which increased significantly (*P* < 0.001) postoperatively to 38.3 (SD 10.8) and 43 (IQR 12). The mean and median and preoperative Tegner score was 1.8 (SD 1) and 2 (IQR 2) which increased significantly (*P* = 0.02) to 2.4 (SD 1.4) and 2 (IQR 2) postoperatively. The mean and median preoperative AKSS O was 54.1 (SD 13.8) and 54 (IQR 19) and increased significantly (*P* < 0.001) to 87.5 (SD 16.3) and 93 (IQR 10) postoperatively. The mean and median preoperative AKSS F was 63.6 (SD 14.6) and 62.5 (IQR 15) which increased significantly (*P* < 0.001) to 74.8 (SD 24.3) and 80 (IQR 40) postoperatively.

Sixty-four patients had no significant event of interest during the duration of the study. There were four reoperations during the study which included two revisions. The reoperations included two arthroscopies (one for pain and the other for a torn lateral meniscus) and two revisions, one of which was a conversion to TKR following a lateral tibial plateau fracture following a traumatic fall and a bearing exchange for a bearing dislocation. There were two deaths unrelated to surgery.

### Comparison of the normal, osteopenic and osteoporotic groups

There were no significant differences in the postoperative OKS, OKS function subscales, OKS pain subscales, and Tegner and AKSS-O scores of the normal, osteopenic and osteoporotic groups (Table 3). There was a significant difference (*P* = 0.04) between in the postoperative AKSS-F between the normal (mean = 78.4, median = 90) and osteoporotic groups (mean = 60.5, median = 65). The osteoporotic group had a lower AKSS-F than the osteopenic (mean 77.8, median 80) group, but this did not reach statistical significance (*P* = 0.07). No differences (*P* = 0.82) existed in AKSS-F between the osteopenic and normal group.

**Table 3:** Comparisons of PROM scores taken at a mean of 4 years postoperatively. The Kruskal-Wallis test was used to test for significant differences between groups *Post hoc testing revealed significant differences between normal and osteoporotic bone group's AKSS-F scores (*P*=0.04)

Parameter	Normal bone	Osteopenic bone	Osteoporotic bone	Differences between groups for parameter
Mean and median OKS	37 (SD 12.1), 42.5 (IQR 14)	38.9 (SD 10), 43 (IQR 9)	38.5 (SD 11.5), 42.0 (IQR 17)	*P* = 0.83
Mean and median OKS functional subscale score	14.6 (SD 5), 17 (IQR 7.5)	15.5 (SD 4.3), 17 (IQR 6)	14.8 (SD 5.4), 16.5 (IQR 9)	*P* = 0.79
Mean and median OKS pain subscale score	22.4 (SD 7.5), 26.5 (IQR 8)	23.4 (SD 6.4), 26 (IQR 7)	23.4 (SD 6.3), 26 (IQR 7)	*P* = 0.93
Mean and median Tegner	2.9 (SD 1.8), 3 (IQR 2)	2.4 (SD 1.2), 2.5 (IQR 2)	1.7 (SD 1.3), 2 (IQR 1)	*P* = 0.17
Mean and median AKSS-O	89.6 (SD 13.2), 95 (IQR 20)	85.9 (SD 19), 93 (IQR 7)	90 (SD 7.1), 93 (IQR 6)	*P* = 0.67
Mean and median AKSS-F	78.4 (SD 29), 90 (IQR 37.5)	77.8 (SD 20.4), 80 (IQR 35)	60.5 (SD 25.3), 65 (IQR 35)	*P* = 0.07*

In the normal bone group (*n* = 20), there was one reoperation (5%) which also classified as a revision (5%) at 2.2 years follow-up. A 57-year-old female with a BMI of 37.4 fell awkwardly onto a wet surface and sustained a lateral tibial plateau fracture which required conversion to a TKR with a stemmed tibial component. There were no deaths in this group.

In the osteopenic bone group (*n* = 38), there were three reoperations (7.9%) which included one revision (2.6%). The three reoperations were as follows: one arthroscopy in a 61-year-old female at 2.4 years post-operatively for pain with no significant intraoperative findings, one arthroscopy in a 58-year-old female at 4.8 years post-operatively for a lateral meniscal tear with intraoperative findings of a torn discoid lateral meniscus with haemarthrosis, and one bearing exchange in a 42-year-old female at 1.5 years post-operatively following anterior bearing dislocation with intraoperative findings of the bearing in the suprapatellar pouch. Two patients died in the osteopenic group during the study at 2.8 and 4.5 years post-operatively from causes not related to the implant (2.6%).

In the osteoporotic group (*n* = 12), there were no reoperations (0%), revisions (0%) or deaths (0%) during the duration of the study.

## Discussion

This is the first study to report the midterm outcomes of knee replacement surgery in patients with reduced BMD according to the WHO criteria [16]. The main finding of the study was that there was no increased revision or reoperation rate or pain following cementless UKR in patients with osteopenia or osteoporosis compared to controls with normal BMD. This suggests that osteopenia and osteoporosis should not be considered to be a contraindication to cementless Oxford UKR.

After the initial assessment of the cementless Oxford UKR, the surgeons involved with this study used cementless rather than cemented fixation for all medial UKR so as to determine if there was a subgroup of patients in which cementless fixation was not appropriate. Osteopenia and osteoporosis were not considered to be contra-indications, and BMD was not part of the pre-operative assessment. Furthermore, even if at operation the bone was found to be soft or the tibial component did not feel solid, cementless fixation was used. Independent of the UKR, if patients were considered to be at risk of fragility fractures, their general practitioners arranged BMD assessments. We identified cementless UKR with BMD assessments and subdivided these into normal, osteopenia and osteoporosis.

At mean follow-up of 5 years, there were no significant differences in the prevalence of reoperation or revision surgery between the groups. Overall, there was a trend towards decreasing revision and reoperation rates with decreasing BMD, with the revision rate in the normal, osteopenic and osteoporotic groups being respectively 5%, 2.6% and 0%. Furthermore, there were no cases with loosening and the only fracture was in the normal bone group, which was a lateral tibial fracture at 2 years due to trauma. As the numbers in the study are relatively small, the study is underpowered to detect small differences in revision and reoperation rate. However, in view of the results, it is unlikely that there is a major problem with, for example, high loosening or fracture rates associated with decreased BMD.

There were no significant differences in post-operative outcome scores between the groups for OKS, including functional and pain subscales, Tegner and AKSS-O, which includes an assessment of pain. However, the osteoporotic group had a significantly lower (*P* = 0.04) AKSS-F compared to the normal bone group. The AKSS-F score does not assess pain, so we can conclude that osteopenia and osteoporosis does not compromise the improvement in pain achieved by cementless UKR.

It is not clear whether there is a compromise in function associated with osteoporosis. Although the AKSS-F was significantly lower with osteoporosis than the normal bone, the OKS functional subscale was not significantly different. Therefore, the AKSS-F finding could be a result of multiple testing (type 2 error) or it could be because the functional assessment is different from that of the OKS. The OKS functional subscale [17] asks patients questions about how their knee affects their ability to do everyday activities such as washing, getting into and out of a car, kneeling and going down stairs. In contrast, the AKSS-F [18] asks about the absolute distance a patient can walk, the mechanism of mobilisation up and down the stairs and whether they use mobility aids. Patients with a diagnosis of osteoporosis are more likely to be suffering from undiagnosed fragility fractures and other cardiorespiratory morbidities than those with the normal bone. These co-morbidities are probably more likely to affect their absolute walking distance and mechanisms of mobilising [22–24], rather than the compromise in everyday activities due to the knee. Therefore, even if there is a compromise in function with osteoporosis, it is unlikely to be related to cementless fixation of the UKR.

It is perhaps surprising that osteoporosis and osteopenia do not compromise the outcome of cementless medial mobile bearing UKR. The likely explanation is that patients with medial arthritis have varus in the knee and often pre-existing tibia vara. As a result, they will have increased loading through the medial compartment of the knee, which will preserve bone quality medially allowing cementless fixation, even if there is a central decrease in BMD [25–27]. Furthermore, with the mobile bearing, the loads on the bone-implant interface are predominantly compressive which is ideal for cementless fixation [28]. Studies of the Oxford knee have shown that the BMD of proximal tibia is preserved after the operation [29, 30]. In contrast following TKR, the BMD can reduce by up to 57% following surgery [31]. Therefore, the finding that it is safe to use cementless fixation for the Oxford knee in osteoporosis or osteopenia may not apply to other implants.

One of the main strengths of the study is that all patients had a central DEXA scan and we did not just assess the peri-prosthetic bone like previous studies [12, 32]. There is a general consensus that the most appropriate regions for assessing patients for osteopenia and osteoporosis is by using central DEXA measurements from the spine and hip given these conditions are systemic [33, 34].

The study does have its limitations: the study was small with 40 patients having osteopenia or osteoporosis and was therefore underpowered for failure as an endpoint. To undertake an appropriately powered study to identify a small increased risk of failure in osteoporosis would be very difficult as it would require many thousands of patients, and many of these would have to be screened for BMD. Another limitation, as discussed above, was that we only assessed patients that had DEXA scans rather than doing DEXA scans in all cementless cases, so there is potential for selection bias [13]. Additionally, our study did not have radiographic follow-up. Finally, we did not have full information on whether patients diagnosed with clinically significant reduced BMD were commenced on medical treatment, but given adherence to medical therapy is widely reported as being poor [35, 36]; we would not expect this to influence our results.

## Conclusions

In conclusion, we found that patients with osteopenia and osteoporosis can safely have cementless Oxford UKR, and following surgery had similar outcome scores to those with the normal bone. We recognise that our study is small and that larger studies are needed to confirm our findings. Therefore, until these are done, surgeons should be cautious with cementless UKR fixation in osteoporotic bone, even though the evidence suggests it is safe.

## Data Availability

The datasets analysed during the current study are not publicly available due to patient confidentiality.

## Abbreviations

AKSS-F: American Knee Society Score-Functional
AKSS-O: American Knee Society Score-Objective
BMD: Bone mineral density
DEXA: Dual-energy X-ray absorptiometry
OKS: Oxford Knee Score
PROM: Patient-reported outcome measure
TKR: Total knee replacement
UKR: Unicompartmental knee replacement
WHO: World Health Organization
